# Psychometrics in experimental psychology: A case for calibration

**DOI:** 10.3758/s13423-023-02421-z

**Published:** 2023-12-26

**Authors:** Dominik R. Bach

**Affiliations:** 1 Hertz Chair for Artificial Intelligence and Neuroscience, Transdisciplinary Research Area “Life and Health”, University of Bonn, Bonn, Germany; 2 Wellcome Centre for Human Neuroimaging, UCL Queen Square Institute of Neurology, University College London, London, UK

**Keywords:** Calibration, Retrodictive validity, Metrology, Psychometrics, Measurement accuracy, Measurement uncertainty

## Abstract

Psychometrics is historically grounded in the study of individual differences. Consequently, common metrics such as quantitative validity and reliability require between-person variance in a psychological variable to be meaningful. Experimental psychology, in contrast, deals with variance between treatments, and experiments often strive to minimise within-group person variance. In this article, I ask whether and how psychometric evaluation can be performed in experimental psychology. A commonly used strategy is to harness between-person variance in the treatment effect. Using simulated data, I show that this approach can be misleading when between-person variance is low, and in the face of methods variance. I argue that this situation is common in experimental psychology, because low between-person variance is desirable, and because methods variance is no more problematic in experimental settings than any other source of between-person variance. By relating validity and reliability with the corresponding concepts in measurement science outside psychology, I show how experiment-based calibration can serve to compare the psychometric quality of different measurement methods in experimental psychology.

## Introduction

Many psychological theories invoke latent variables^1^ that are not directly accessible, such as memory, attention or confidence. To empirically assess such theories, latent variables must be measured via an observable variable. This raises a question of validity: does the measurement actually tap into the latent variable, and if yes, how well?

Validation of a measurement is an iterative process and generally based on multiple sources (AERA, 2014; Kane, 2016; Messick, 1987). For a historical example from experimental psychology, Pavlov’s work in dogs inspired learning researchers to probe the existence of classical conditioning in humans. On a purely behavioural level, they established that coupling a conditioned stimulus (CS), such as a neutral tone, with an unconditioned stimulus (US), such as an electric shock, would lead to a temporary increase in skin conductance after CS presentation. It was also established that the presence of the CS leads to an increase of the startle reflex elicited by a sudden loud sound. Later on, theories emerged on the cognitive processes that mediate these effects, and a latent variable “CS-US association” was posited, which was thought to underlie the observed behavioural effects in skin conductance response (SCR) and fear-potentiated startle (FPS). In turn, these two (and several other) observables were now used to test ever-more sophisticated theories about how the “CS-US association” comes about. In such experiments, some manipulation is performed during learning, and the strength of the resulting “CS-US association” is then “operationalized as”, or “measured by”, for example, SCR amplitude. At this stage, one might start asking “how well” SCR and FPS measure the latent CS-US association, or whether a new observable – say, heart rate – also measures the CS-US association.

These are the classical questions of validity theory, and the issue of “how well” motivates evaluating quantitative metrics as part of a validity argument (AERA, 2014). Such metrics include convergent validity (the correlation between two different measures of the same latent variable), discriminant validity (the correlation between two measures that supposedly measure different latent variables), and reliability (the correlation between repetitions of the same measure, or between two very similar measures termed “parallel tests”). Similar questions arise in the evaluation of measurement methods throughout psychology, from questionnaires and attainment tests to reaction times and physiological indices of cognitive processes. These measurement methods often involve some transformation of the raw observed data to produce a summary measure, such as a questionnaire sum score, a reaction time score after outlier rejection, or a peak score of a continuous physiological variable.

In this article, I argue that formal validation by way of quantitative metrics is common in the study of individual differences but much more infrequent in experimental psychology, which often investigates general rules of the mind. Using simulated data, I discuss the problems associated with using classical psychometric coefficients for evaluating the measurement of general treatment effects. After highlighting the challenge and several situations that lead to seemingly paradoxical psychometric coefficients, I derive an alternative metric, "retrodictive validity”, from the concept of experiment-based calibration in the field of measurement science (Phillips et al., 2001). As I show in detail, the concept of experiment-based calibration, originally conceived in physics, sits naturally with experimental psychology. It takes an experimental manipulation (i.e., CS-US coupling) as a starting point, and assesses how well a measurement reproduces the effect of this experimental manipulation. In essence, thus, it constitutes a form of criterion validity with the experimental manipulation as criterion.

## Classical psychometrics

Cronbach famously divided psychology into correlational and experimental, where correlational psychology seeks to explain *variance between persons* (i.e., how and why persons differ from one another), and experimental psychology seeks to explain *variance induced by experimental treatments* (i.e., why the effect of an experimental treatment is different from a baseline, or from the effect of other treatments) (Cronbach, 1957). There is now overlap and crosstalk between the approaches (Hedge et al., 2018), partly generated by an interest in explaining between-person variance of experimental treatment effects (Rouder & Haaf, 2019). Examples include cognitive control in various experimental tasks and how it relates to other personality traits including psychopathology (Bastian et al., 2022), or the development of clinical-diagnostic anxiety tests based on experimental tasks (Bach, 2022). However, a substantial division remains between the study of between-person variance, and the study of general rules of psychology in an experimental approach (Borsboom et al., 2009), which I term “general experimental psychology” throughout this article.

Measurement validation seems necessary in all branches of psychology that deal with latent variables. However, validation theory, as part of the wider field of psychometrics, is firmly grounded in the study of between-person variance (Borsboom et al., 2009). This has important consequences for the evaluation of measurement methods: all psychometric coefficients used for validation are based on concepts that harness and require between-person variance (McDonald, 2013). In contrast, general experimental psychology regards incidental between-person variance (within each treatment group) as error. In turn, many experimenters strive to minimise (within-group) person variance (Hedge et al., 2018). How can measurement methods then be evaluated in these fields? Suppose an experimenter runs a classical conditioning experiment in which CS and US are coupled, and seeks to validate FPS as a measure of CS-US association. In line with the demands of experimental design, the researcher will seek to ensure that the baseline association (before learning) is uniformly small, with little or no variance between individuals. In this situation, how can psychometric properties be assessed?

Importantly, the role of treatment variance in experimental psychology is not symmetric to the role of between-person variance in correlational psychology; their roles cannot simply be reversed. Correlational psychology deals with variance between many persons under one constant (that is, no) experimental treatment. The reverse – many treatments in one person – is not the usual approach in experimental psychology; instead, it deals with variance between several (often only a few) treatments in many persons. Hence, it is plausible to assume that there is between-treatment and between-person variance in an experimental study. The latter can then be harnessed for measurement validation. To this end, two approaches are currently used.

The first is to assume that the variable in question differs between persons under baseline (no treatment) conditions, and to harness this baseline between-person variance. For example, sleepiness might be different between persons as well as between treatments. To evaluate a sleepiness instrument, validity and reliability assessment can be based on between-person variance in the absence of experimental treatment; and one may assume that this instrument is then also valid to assess treatment effects.

While this approach is plausible for some variables, it can be difficult to apply or implausible for others. One example are variables which – outside an experimental treatment – are unstable and fluctuate rapidly, such as momentary spatial attention. This would make the design of parallel tests to assess reliability rather challenging. Another example is variables for which the measurement appears meaningless outside an experimental manipulation, such as declarative item memory. Under baseline conditions – before having seen the item list – people may uniformly respond that they cannot recall something they have not yet seen. In such examples, validating the measurement method under baseline (no treatment) conditions would be difficult to substantiate.

A second, intuitively plausible and frequently used approach is to harness incidental between-person variance in the effect of a within-subject treatment (for examples from associative learning, see Fredrikson et al., 1993; Torrents-Rodas et al., 2014; Zeidan et al., 2012). Explaining between-person variance in the treatment effect is sometimes the main focus of research (Bastian et al., 2022; Hedge et al., 2018; Rouder & Haaf, 2019; Schuch et al., 2022). In these cases, the approach is firmly grounded in psychometric theory. Here, I ask whether the approach is also useful when the focus of research is on general treatment effects.

**Fig. 1:**
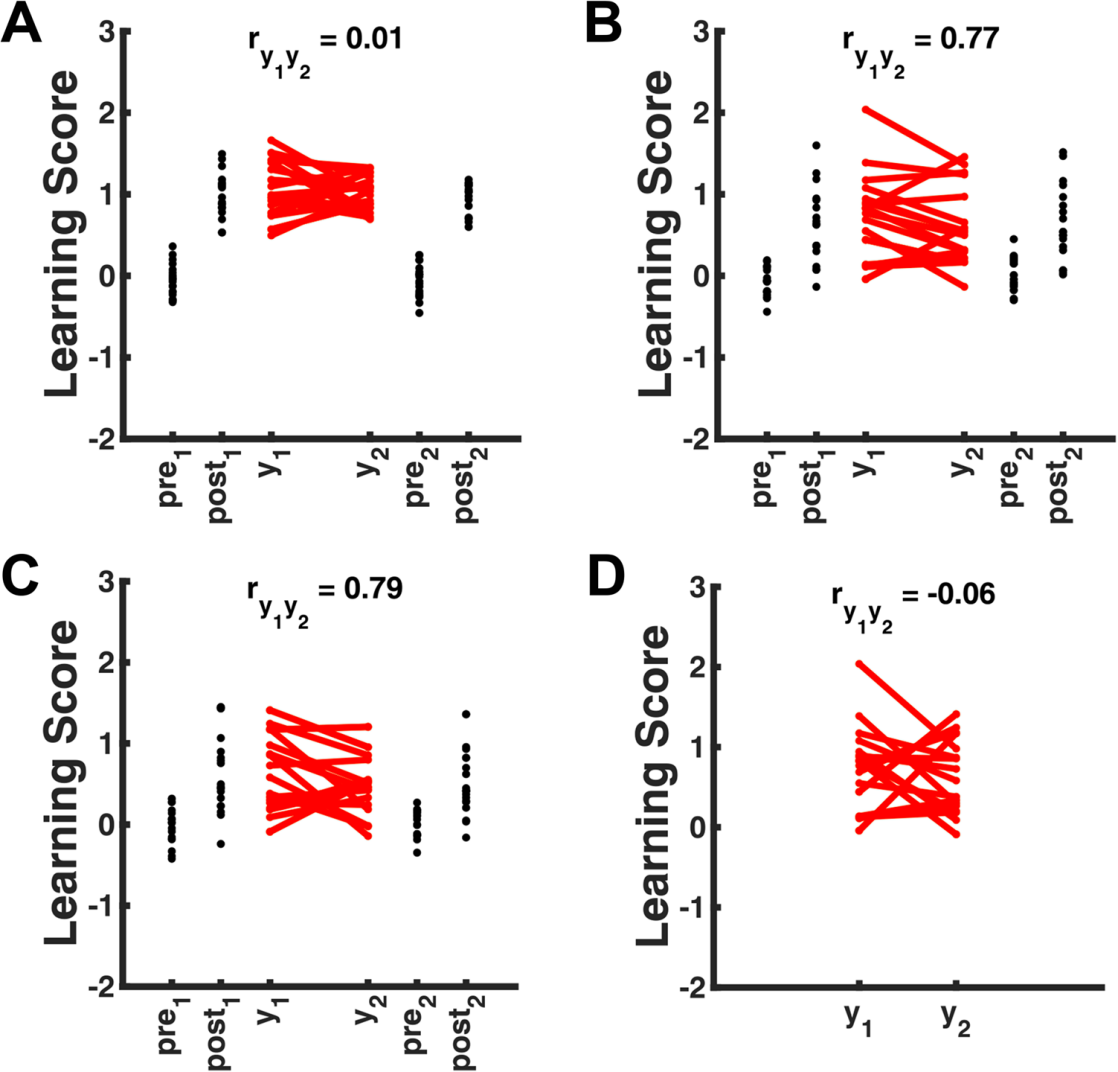
Three examples show how classical psychometric coefficients can appear paradoxical when evaluating the measurement of general treatment effects. Panels **A**–**C** show results from simulated within-subjects experiments with an assessment before (pre) and after (post) learning, as well as the respective differences, i.e., change scores (y), which are to be evaluated. For each panel, the left-hand side shows results from Experiment 1, and the right-hand side results from Experiment 2 (a supposedly parallel test) with the same participants. **A**: Learning score derived from FPS. High (standardised) effect size in differentiating the experimental treatments, but low between-subjects variance in the true score leads to low reliability coefficient. **B**: Learning score derived from SCR. Low effect size in differentiating the experimental treatments, but high between-subjects variance in a multiplicative methods effect in the data-generating process leads to high reliability coefficient. **C**: Learning score derived from heart rate. Measure with similar properties as in B, but suffering from a different and uncorrelated methods effect. **D**: Direct comparison of the learning scores from B and C (first assessment). Because they suffer from uncorrelated methods effects, convergent validity coefficient between the measures is low. r_y1y2_: correlation between the change scores from two experiments (i.e., test-retest reliability coefficient, panels **A**–**C**) or two measures within the same experiment (i.e., convergent validity coefficient, panel **D**)

For example, one can expand the aforementioned validation study by including a second conditioning experiment with the same individuals and a different CS-US pair, which might be regarded as a parallel test for the assessment of test-retest reliability. Under the implicit or explicit assumption that there exists a somewhat stable propensity (across experiments) to acquire a CS-US association, test-retest reliability yields information on measurement precision. However, the heuristic plausibility of this approach obscures substantial problems, which are illustrated in Fig. 1 and discussed in the next section. All of these problems constitute well-known challenges in psychometrics and correlational psychology (McDonald, 2013). What makes them special in experimental psychology is that they are not challenging for experimental research as such, only for the interpretation of psychometric coefficients – and thus, there might be many common situations in experimental psychology where current validation methods cannot be applied.

## Examples

The following examples are based on simulations. Mean learning scores before and after a learning experiment were coded as [0, 1]. Simulated true scores had no within-condition variability. Random measurement error was drawn from a normal distribution with the same variance across measurement methods (*σ* = .05). Person-specific methods effects were drawn from a half-normal distribution with the same variance (*σ* = 1), and were multiplied with the true scores before adding observation noise. Correlation coefficients are based on N = 100 simulated participants. Figure 1 shows N = 20 randomly selected participants for each experiment to improve visualisation. The code for reproducing the simulations and figure is available via the Open Science Framework (https://osf.io/3zkvh/).

## Example 1: Reliability and convergent validity can be meaningless if true score variance is low

In the first example (Fig. 1A), the experimenters run two conditioning experiments with the same individuals in order to assess test-retest reliability of FPS. For each person and for either experiment, they compute a change score as the pre-post difference in FPS. As Fig. 1A shows, FPS is larger after than before learning, in both experiments. The change scores (*y*_*1*_ and *y*_*2*_) are positive for all individuals and relatively far away from zero, meaning that they have a large (standardised) effect size.^2^ Heuristically, many experimenters would conclude that FPS is a good measure to "tap into" the CS-US association. As an anonymous reviewer of this article pointed out, one might say it has good “psychonomic properties”.

However, when researchers then compute test-retest reliability, i.e., the correlation between *y*_*1*_ and *y*_*2*_, it is close to zero (${r}_{y_1{y}_2}=0.01$). This seems to indicate that the measurement method has bad “psychometric properties“, and should not be used.

To resolve the paradox, in this hypothetical example there is no variance in the assumed stable learning propensity, and all of the variability between persons is due to random error in the measurement. Statistically,^3^ if we denote variance of the (population) change score with ${\sigma}_T^2$, the (population) variance of the two change score measures *Y*_1_ and *Y*_2_ with ${\sigma}_{Y_1}^2$ and ${\sigma}_{Y_2}^2$ , respectively, and their (population) reliability coefficient with ${\rho}_{Y_1{Y}_2}$, then it can be shown (McDonald, 2013) that under plausible assumptions:

$${\rho}_{Y_1{Y}_2}=\frac{\sigma_T^2}{\sigma_{Y_1}^2}=\frac{\sigma_T^2}{\sigma_{Y_2}^2}.$$

Hence, if ${\sigma}_T^2\ll {\sigma}_Y^2$, then ${\rho}_{Y_1{Y}_2}\cong 0$, or in words: if between-person variance in the true change score is much smaller than random error in the measured score, then reliability will be close to zero. This precludes interpreting the sample reliability coefficient on its own as reflecting on the quality of the measurement method. This is a realistic scenario in experimental psychology, which often strives to minimise between-person variance relative to treatment variance (Hedge et al., 2018). In contrast, it is not a typical case in correlational psychology, where attributes with low between-person variance are rarely an interesting topic of investigation. Drawing on psychometric theory, one can still derive an estimate of the measurement error variance ${\sigma}_E^2$, under plausible assumptions, by the formula (McDonald, 2013):

$${\sigma}_E^2=\left(1-{\rho}_{Y_1{Y}_2}\right){\sigma}_{Y_1}^2,$$

and this can provide a useful insight into properties of the measurement instrument. However, I demonstrate below how to assess measurement error in a simpler way with a single test, rather than with two parallel tests.

Notably, the same problem arises for convergent validity coefficients. Imagine the second set of scores *y*_2_ not coming from a different experiment with FPS, but from the same experiment with a different measure (e.g., declarative ratings of the CS-US contingency). Then, correlation between the two measures constitutes a convergent validity coefficient, which is supposed to be high if both measures tap into the same latent variable. Again, convergent validity will necessarily be low when true score variance is low. In this case, classical psychometrics would regard both measures as having low validity, i.e., they are not truthful measures of the CS-US association. This, however, is at odds with their strong relation to the experimental manipulation.

## Example 2: High reliability can be meaningless in the presence of methods variance

In this example, the researchers perform the experiments described above, in order to test a measure of the CS-US association based on SCR. They compare this to FPS, which has the properties described in Example 1. The experimenters find that the SCR-based measure has a much higher reliability coefficient than the FPS measure (Fig. 1A, B). Should we believe that it is a more useful measure of associative learning? In fact, as Fig. 1B shows, the effect sizes of the SCR-derived scores in either of the experiments are much lower than for the FPS measure, which would prompt many experimental psychologists to conclude that it is not such a good measure. How can this discrepancy be resolved?

Many measurements in (correlational and experimental) psychology can be influenced by between-person differences in latent variables that are unrelated to the variable of interest. In psychometrics, this is often termed *construct-irrelevant variance*, and a common case is methods variance. For example, language abilities are assessed via a test that also requires motivation. Persons who are generally more motivated will have higher scores, but they will also have higher scores in a test of mathematics that measures a different latent variable. Similarly, personality traits are assessed via a questionnaire that requires meta-cognitive abilities, and CS-US association is assessed by measuring the response of the autonomic nervous system. Between-person variance in the measured values might be due to variance in the latent variable, but also due to method-specific variance in the way the attribute is expressed in behaviour. In other words, persons might systematically differ in how they express a CS-US association in the autonomic nervous system, and this variability might be stable over repeated tests, while the values of the CS-US association might not. In these cases, reliability coefficients are misleading. The simulated example includes a multiplicative methods effect in the data-generating process, i.e., the transformation from true score to measured score includes a scaling that is systematically different between persons. This can be seen from the lower variability in the pre- compared to the post-treatment condition, and constitutes a typical source of noise in the assessment of peripheral autonomic indices.

This problem is well recognised in psychometrics and can be detected by assessing discriminant validity, the correlation between two similarly designed tests for different latent variables. For example, the correlation between a language and a mathematics test that use a similar method (discriminant validity) should be lower than the correlation between two language tests that use different methods (convergent validity). While this validation approach is widespread and mature in the context of multi-trait, multi-methods matrix evalution for individual differences (Campbell & Fiske, 1959; Eid & Nussbeck, 2009; Widaman, 1985), it is rarely used in experimental psychology. A potential reason is that methods variance is problematic for interpreting validity coefficients and for assessing individual differences, but not for measurement of general treatment effects. For assessing general treatment effects, any between-subject variability is nuisance. There is no conceptual distinction between variability in treatment effects on the latent attribute and variability in expressing that latent attribute in behaviour. Both of these are undesirable, and many experimental psychologists will seek to minimise variability of both sorts, rather than spend resources to distinguish them.

## Example 3: Low convergent validity can be meaningless in the presence of methods variance

In this example, the experimenters introduce a third measure of associative learning, derived from heart rate. As illustrated in Fig. 1C, the effect sizes and reliability coefficients in each of the two experiments are comparable to the SCR-derived measure shown in Fig. 1B. However, the correlation between the two measures is close to zero, i.e., they have low convergent validity (Fig. 1D). A naïve psychometric view would hold that at least one of the two measures does not measure associative learning. Or perhaps, that they measure two different forms of associative learning which one might call (with some physiological insight) "sympathetic conditioning" and "parasympathetic conditioning".

However, in this hypothetical example, both measures are indicative of the same latent attribute (which has low variability) – but each of them is contaminated by independent methods effects with high between-subjects variability. As in Example 2, such methods variance can be detected in a multi-trait, multi-method maxtrix approach – but this is costly and might be pointless if the goal is to optimise the measurement of general treatment effects.

### Synopsis of the examples

The common theme of preceding examples was that they harness between-person variance in treatment effects, to evaluate the quality of general treatment-effect measurement. This approach can lead to erroneous conclusions in cases where between-subjects variance of the treatment effect is low, or is caused by methods variance. While these cases might be detected in a classical psychometric framework with multi-trait multi-methods matrix assessments, these are expensive and time-consuming, and might ultimately have little relevance for evaluating the measurement of general treatment effects: low between-subject variance is desirable in general experimental psychology, and methods variance is a nuisance but no more problematic than any other source of between-subject variance. In summary, a suitable psychometric evaluation of general experimental treatment effects should be applicable in the presence of low true-score variance and methods variance.

## Requirements for psychometric assessment in general experimental psychology

To identify further requirements for a suitable psychometric assessment in experimental psychology, it is helpful to link psychometrics with metrology (i.e., measurement science) outside psychology. Measurement science stipulates that any measurement should be accurate, and distinguishes two components of accuracy (BIPM, 2012). These are well known in psychology but under diverging labels, and so I recapitulate them in brief.

The first accuracy component is trueness (BIPM, 2012). A measurement is truthful if for each value of the true score, the expected value of the measured value is close to the true score, i.e., systematic measurement error (also termed bias) is low. From a classical psychometric perspective, this definition might seem surprising, because classical true score theory simply defines the true score as the expected value of the measured score (McDonald, 2013; Novick, 1966), such that there can be no bias. In this definition, which one might call “empiricist”, the true score depends on the measurement method, and two measurement methods for the same latent variable will lead to different true scores. In contrast, metrology (and some areas of contemporary of psychometrics) define a “realist” true score as an abstract or mathematical object, which is independent from any particular measurement.^4^ However, even in classical true score theory, there is a possibility that the empiricist true score does not reflect the latent variable one is interested in, and this is embodied in the concept of validity. Quantitative validity coefficients (Campbell & Fiske, 1959; Fornell & Larcker, 1981; McDonald, 2013; Widaman, 1985) constitute an important source of evidence for a validity argument (Cronbach & Meehl, 1955; Kane, 2016; Messick, 1987). However, as exemplified above, the problem with quantitative validity coefficients is twofold: first, they require true score variance (Example 1), and secondly, they are difficult to interpret in the presence of (large) methods variance (Example 3). Hence, they don’t fulfil the requirements for the evaluation of trueness in general experimental psychology.

The second accuracy component is precision (BIPM, 2012). A measurement is precise if, for each constant value of the true score, repeated measurements yield similar measured values. In other words, precision is the inverse of random measurement error variance. In psychometrics, measurement error can be assessed via the concept of reliability. We have seen that reliability coefficients are not meaningful if true score variance is low, or in the presence of methods variance. In the first (but not in the second) case, measurement error variance can still be calculated from reliability coefficients (McDonald, 2013), and can then be compared between different measurement methods. However, as a method for evaluating precision in general experimental psychology, this may be overly complicated, because it requires parallel (or repeated) tests. In fact, there are arguably experimental situations in which repeating a measurement is not even an option.

## The calibration concept

In the natural sciences, measurement quality is evaluated by comparing measured scores to a reference; a process termed *calibration* (Phillips et al., 2001). This reference (although not fully accurate itself) provides the predicted values for the measurement. The best possible reference is termed a *standard*. Traditionally, these standards were often material – such as the international prototype kilogram (a block of metal) or metre (a metal rod) stored in Paris. Obviously, material standards cannot exist in psychology (Krantz et al., 1971). Since the 1960s, however, metrology in the natural sciences has shifted towards experiment-based calibration. Here, measurements are compared against the outcomes of a carefully designed experiment that prescribes standard measured scores. This concept sits well within experimental psychology, which is focused on using experimental treatments to manipulate psychological variables. Arguably, psychological theories are often less well equipped to predict the latent variable in an experiment very precisely. In some fields, latent variables may at best be predicted on an ordinal level, for example, one may assume that more learning trials lead to higher memory scores but do not necessarily formulate a (general) numerical relation between the number of trials and the learning score. In other fields, numerical (“interval-scale”) predictions are already possible, for example, predicting perceived lightness (the standard scores of the attribute) from physical lightness (the independent variable in the experiment) together with the CIECAM02 model (a numerical psychological theory of visual perception used in industry settings). Even here, there may still be a deviation of the actually generated true score on the same order of magnitude as the measurement error. Notwithstanding, previous work has statistically demonstrated that calibration can distinguish accuracy of two measurement methods even in the presence of imprecise experimental methods, as long as there is a positive (not necessarily high) correlation between the predicted and the achieved (true) scores of the latent variable (Bach et al., 2020). Then, the correlation between predicted and measured scores, termed retrodictive validity, depends jointly on random and systematic measurement error and, thus, on trueness and precision. This means that ranking different measurement methods by their retrodictive validity ranks them by measurement error. From the perspective of experimental research, the relation between predicted and measured attribute scores is often termed “effect size”. However, this term is most often used in the context of substantive, hypothesis-testing research. In the calibration approach, the effect of the experimental manipulation is assumed a priori, rather than hypothesised and tested. This is why I prefer the term “retrodictive validity” (Bach & Melinscak, 2020) in this context, which acknowledges that it is a form of criterion validity where the criterion is experimentally defined (Messick, 1987), rather than a measured variable as in concurrent or predictive validity assessments (McDonald, 2013).

To summarise, the concept of experiment-based calibration is to compare a set of measured scores to the predicted outcomes of a well-established experiment. This appears feasible in experimental psychology and does not require any between-person variance in the measured attribute. It is thus not vulnerable to the problems highlighted in the examples, and it does not require parallel tests.

## Calibration: Practical implementation

**Fig. 2:**
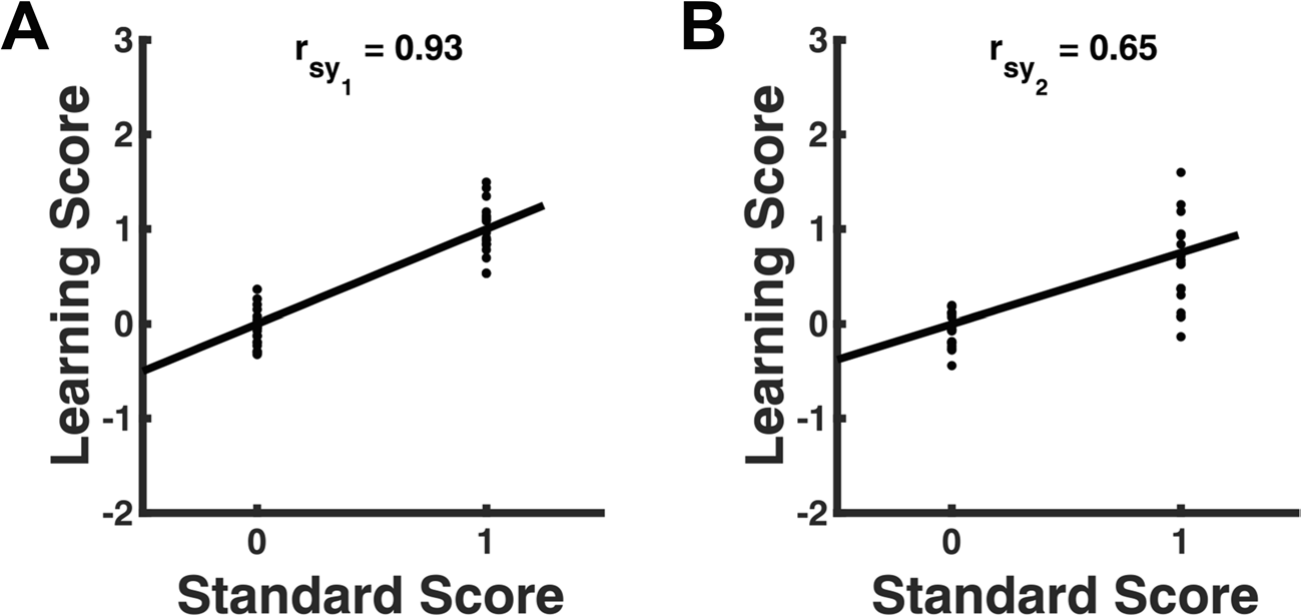
Simulated calibration experiment, in which the measurement of CS-US association (learning score) is evaluated for two methods, one based on pupil size (panel** A**) and one based on SCR (panel** B**). The panels show the predicted standard scores (dummy-coded as 0 before learning, and 1 after learning), and the resulting learning score for N = 20 subjects. A regression line is fitted for visualisation, and retrodictive validity is shown as correlation between standard and learning scores (r_sy_). Retrodictive validity is higher for the pupil size measure, because it has lower measurement error, i.e., the measured values are closer to the true scores

To demonstrate the practical implementation of a calibration concept, we can run a new conditioning experiment, in which SCR and a new measure of learning, based on pupil size, are measured concurrently (Fig. 2). In this hypothetical example, one of the two measures (SCR) is imbued with irrelevant methods variance, and the true score variance is negligible. We have seen before that this is a challenging situation for classical psychometric coefficients. Here, we ask whether calibration results in a more useful comparison of the two methods.

As typical in the calibration approach, we take it for granted that the conditioning procedure induces a CS-US association; this learned association constitutes the latent attribute in question. To define the predicted (standard) values of the latent attribute, we simply use “dummy” values of 0 (before) and 1 (after), because there are just two levels of experimental treatment, and because the scaling of psychological attributes is arbitrary. (If there were more than two levels then of course the actual values would matter.) Figure 2 shows the outcomes for the two measures. It is easy to see that the correlation between the standard values, and the measured values, is much higher for pupil size (panel A) than SCR (panel B). This captures the intuition that SCR is much more variable, and the learning scores before and after learning are highly overlapping in the group. In this simulated example, higher retrodictive validity for pupil size emerges because (random) measurement error for pupil size after learning is much smaller than for SCR. This seems to confirm that calibration is a useful concept to compare measurement methods in the absence of true score variance and in the presence of methods variance. Furthermore, the difference between the two methods in retrodictive validity can be established without running parallel or repeated tests. This offers an advantage over classical psychometric concepts in terms of resource effectiveness, because it requires only one dedicated calibration experiment and not two.

To summarise, a calibration experiment proceeds in three steps:

1. Select an experimental procedure that uncontroversially affects the psychological variable to be meausured. This may not be possible in all fields of general experimental psychology, but there are many fields where at least ordinal predictions for the expected true scores in such experiment can be made (such as “high” and “low”). In some areas of perception and cognitive research, it will even be possible to specify the predicted true scores as numerical values. Importantly, as in any experiment, the procedure should be crafted in a way that does not affect other latent variables, or in other words, that there are no experimental confounds.
2. Perform a dedicated calibration experiment with this procedure, outside of any substantive hypothesis test, and use at least two methods for measuring the psychological attribute in question.
3. Compare retrodictive validity between the two methods; the method with higher retrodictive validity will have lower measurement error variance .
4. This selected method can then be used in further experiments design to test substantive research questions.

It is important to point out that calibration should be performed in dedicated calibration experiments. Furthermore, calibration requires an experimental manipulation that is already known to affect the latent variable in question. Thus, it is ideally suited for consolidated fields of psychology, but cannot be performed on an experiment designed to test whether the treatment has any effect in the first place. It should also not be performed on published experiments that were originally designed to test a substantive hypothesis, because publications are biased towards reporting unambiguous evidence, and this implies that published effect sizes regularly overestimate the true ones (Button et al., 2013; Ioannidis, 2005). This is not a concern if an experiment is dedicated as a validation study.

Importantly, retrodictive validity of two measures always needs to be compared within the same experimental procedure, and ideally within the same experiment. This is because retrodictive validity depends not only on measurement error but also on the effectiveness of the experimental procedure – to what extent the predicted true scores are actually achieved. This is fixed within any calibration experiment, and it might be relatively similar between two experiments performed under the same circumstances and sampling from the same participant population. However, it could be drastically different between two different experimental procedures, and this is why retrodictive validity cannot be compared between two experiments using different procedures.

In turn, because retrodictive validity depends on the measurement method and the effectiveness of the experimental procedure, it can serve as a metric to evaluate the experimental procedure as such, by fixing the measurement method and varying the experimental procedure. In a calibration context, this can help optimise the quality of the calibration results. While an imprecise experimental manipulation does not invalidate the calibration approach (Bach et al., 2020), it can make retrodictive validity coefficients more variable (Bach, 2021). This is undesirable as it might render a comparison of closely related methods inconclusive. Following from steps 1–4 above, one might add the optional following steps to improve the calibration procedure:

5. Perform a dedicated calibration experiment with a different experimental procedure and the same measurement methods.
6. Compare retrodictive validity (for the same measurement method) between the two experimental procedures; the procedure with higher retrodictive validity will have true scores that are more closely related to the standard scores.
7. This procedure can then be used in further calibration experiments.

## Calibration: When is it useful?

Experiment-based calibration yields a useful assessment of measurement error variance in many common cases where classical psychometric concepts are not applicable. Often, it will also be less complicated because there is no need to run parallel or repeated tests (as for establishing reliability) or to find discriminant latent variables. Thus, it might lower the bar for psychometric evaluation of measurement methods in experimental psychology. In the following, I summarise the situations in which calibration can be useful, and what its limitations are.

In general, experiment-based calibration, as implemented in psychology, is meant to compare several measurement methods within a single experiment. This can be helpful when there is a requirement to select a single method out of several existing ones. Crucially, “method” here refers not only to the type of observation (e.g., reaction time vs. choice vs. verbal report), but also to the way that these observations are summarised into a score. Thus, two different ways of rejecting reaction time outliers constitute two different measurement methods, which can be compared against one another. In fact, there are many ways of transforming raw reaction times into a dependent variable (Simmons et al., 2011), and it has been suggested that selecting a method post hoc might add to problematic research practices that reduce replicability of research findings (Simmons et al., 2011). Experiment-based calibration offers a rational and independent criterion to select one measurement method a priori, for example for the purpose of pre-registration. Thus, it could potentially have widespread application throughout experimental psychology. Of course, if there was only one plausible method for acquiring and analysing data in a specific situation, then calibration would be of little use.

On the other hand, in order to be successful, experiment-based calibration has some requirements. First, calibration requires that an experimental manipulation is known to affect the latent variable in question. Crucially, it is not required that consensus exists on *how to measure* the latent variable, or what its *psychological nature* is. For example, there is much disagreement on the nature of a CS-US assocation (Bach et al., 2023), and how to best assess it in humans (Ojala & Bach, 2020). Notwithstanding, there is broad consensus, based on many decades of research, on the type of procedures that generate CS-US associations, as highlighted by a recent consensus design of a calibration experiment (Bach et al., 2023). There are many areas of psychology where a latent variable is known or believed to be affected by some experimental procedures. In the simplest case, for example, in perceptual research, a suitable procedure can simply consist of changing physical attributes, in order to change their percepts as latent variables. Since it is not necessary to precisely know the relation between physical attributes and latent variables, this allows for a broad range of application. Many other variables can be experimentally manipulated across cognitive research, for example, item memory, decision confidence, state and action values, spatial attention, to name just a few.

## Discussion

Classical psychometric concepts were developed in the context of correlational psychology, i.e., the study of incidentally existing differences between individuals. Applying these concepts to evaluate experimental measurement of general treatment effects is problematic for three reasons. First, because their interpretation rests on between-subjects variance, which experimental psychology often seeks to minimise. Secondly, because their interpretation is invalid in the face of methods variance; but methods variance is not problematic for experimental measurement as such. Third, because their assessment is more complex than that of alternative approaches. As one such alternative approach, I present the idea of experiment-based calibration, which is a standard concept in metrology (measurement science) outside psychology. I show how this can be applied in experimental psychology, and how it bypasses some of the pitfalls identified. Ideally, such calibration would be a community effort and supported by community consensus on the procedures used (see, e.g., Bach et al., 2023).

In contrast to calibration in the physical sciences, experiment-based calibration in psychology does not evaluate a single measurement method on its own. Instead, it compares different methods within the same experiment. It is sometimes argued that instead of selecting one particular method for quantification of a psychological variable, it would be desirable to simultaneously employ several methods in an experiment. However, there are many situations where this is not an option. For one, “measurement method” not only refers to observables or data types, but also to different transformations of the same observed data. There are many ways of transforming raw reaction times into a dependent variable (Simmons et al., 2011); researchers will usually only report one of them rather than simultaneously performing statistical analyses on several (highly related) transformations. Such different transformations can easily be compared a priori with experiment-based calibration. Another situation arises from the increasingly common mandate to pre-register observables and data transformations and choose a “primary outcome”. Psychometric coefficients, and experiment-based calibration in particular, can assist these difficult choices (Bach et al., 2020).

Many types of experimental measurement methods are not routinely evaluated by their psychometric properties. This may partly reflect experimenters’ intuition about the problems identified here. Calibration might offer a relatively low-cost means to more rigorously establish the quality of common measurement methods in this field.

## Footnotes

1 Latent variables are also termed “constructs“ or “attributes“, with slightly different nuances. Here, I stick with the label “latent variable“, and refer to the specialist psychometric literature for a discussion of these nuances.

2 With "effect size“, I always refer to the standardised effect size. In this example of a within-subjects difference score, common metrics include Cohen’s d and Hedge’s g.

3 The discussion is based on the classical true score model (McDonald, 2013), noting that analogous questions arise in more contemporary and complex psychometric frameworks.

4 The realist true score can obviously be expressed in different units, for example the true score of the latent variable “temperature“ can be expressed in Celsius or Fahrenheit, which correspond to different linear functions, but this does not change the true score.
